# Prevalence of *Giardia* and *Cryptosporidium* in young livestock and dogs in Magude District of Maputo Province, Mozambique

**DOI:** 10.4102/ojvr.v86i1.1709

**Published:** 2019-08-12

**Authors:** Regina D. Miambo, Benigna Laitela, Mokgadi P. Malatji, Sonia M. de Santana Afonso, Alberto P. Junior, Johan Lindh, Samson Mukaratirwa

**Affiliations:** 1 Department of Para-Clinics, Faculty of Veterinary Medicine, Eduardo Mondlane University, Maputo, Mozambique; 2 School of Life Science, College of Agriculture, Engineering and Science, University of KwaZulu-Natal, Westville, South Africa; 3 Department of Cell and Molecular Biology, Uppsala University, Uppsala, Sweden

**Keywords:** zoonoses, *Giardia*, *Cryptosporidium*, dogs, calves, goats, Mozambique

## Abstract

**Background:**

*Giardia* and *Cryptosporidium* species are significant zoonotic parasites of humans and domesticated animals.

**Objectives:**

The study aimed to determine the prevalence of *Giardia* and *Cryptosporidium* in livestock and dogs of the Magude District.

**Method:**

The flotation technique (Willis), modified Ziehl-Neelsen (mZN) and direct and indirect immunofluorescence (DIF and IIF) techniques were applied to determine the prevalence of *Giardia* and *Cryptosporidium* species in faecal samples of dog pups (156), goat kids (60) and calves (480) from the Magude District of Mozambique from February to September 2015.

**Results:**

Using Willis, IIF and DIF, the prevalence of *Giardia* in calves was 0%, 8.1%, and 6.0%; in dogs 0.6%, 8.3% and 5.7% and for goats 0% and 13.3% (IIF was not performed), respectively. The prevalence of *Cryptosporidium* in calves using Willis, mZN, IIF and DIF was 0%, 3.8%, 4.7% and 0.4% and in dogs 0%, 0.6%, 6.4% and 0.6%, respectively. The parasite was not detected in goats.

**Conclusion:**

Results from the present study showed that IIF performed better diagnosis of *Giardia* and *Cryptosporidium*, and that the mZN can be used as an alternative for *Cryptosporidium* because of the high cost of IIF. There is a need for identification of genotypes or subtypes of these parasites through application of molecular techniques in order to determine their zoonotic potential, and we advocate a ‘one health’ approach in the control and prevention of these parasites.

## Introduction

Protozoan species from the genus *Giardia* and *Cryptosporidium* are known to infect domestic and wild animals (Taylor, Coop & Wall 2007) and are implicated as causative agents of diarrhoea in children, and as opportunistic infections in HIV-positive patients (Fayer, Morgan & Upton 2000; Irisarri-Gutiérrez et al. 2017; Morgan et al. 2000; Pedersen et al. 2014; Sow et al. 2016; Wang et al. 2018). In domestic animals, the parasites are mainly prevalent in neonates and young animals (Baroudi et al. 2018; De Waal 2012; Hamnes et al. 2006) with consequent economic loss because of different levels of morbidity and mortality (De Graaf et al. 1999) particularly when they occur in concomitant infections with helminthic infections (Taylor et al. 2007). There has been a description of two subtypes of *Cryptosporidium parvum* (Baroudi et al. 2018; Fayer et al. 2000; Santana et al. 2018; Squire et al. 2017) and multiple genotypes within the species *Giardia duodenalis* (Ebner et al. 2015; Feng & Xiao 2011; Itagaki et al. 2005; Santín, Trout & Fayer 2007; Sommer et al. 2018), and only a few are of zoonotic significance.

Depending on the purpose of the study, different techniques can be applied for the diagnosis of *Giardia* and *Cryptosporidium*. Direct smears with or without staining and concentration techniques are mainly used routinely in the laboratory, and despite the relatively low cost, they have a disadvantage of low sensitivity (Cheesbrough 1987; De Waal 2012). In view of this limitation, immunological techniques based on the detection of antigens such as enzyme-linked immunosorbent assay (ELISA), the immunofluorescence (IF) staining method and the molecular test polymerase chain reaction (PCR) which detects the parasite deoxyribonucleic acid (DNA) have been applied in epidemiological studies, and they have proved to be more sensitive and specific (Geurden et al. 2008; Gómez-Couso, Méndez-Hermida & Ares-Mazás 2006; Soares & Tasca 2016).

Studies conducted in Mozambique have reported prevalence of 8.1% for *Giardia intestinalis* and 7.1% for *Cryptosporidium* spp. in humans (Irisarri-Gutiérrez et al. 2017). Mixed helminths infections of *Toxocara canis* and *Ancylostoma* spp. in dogs were reported by Cruz and Silva (1971) and Santos, Nhantumbo and Alho (2013); however, there was no reference to *Giardia* and *Cryptosporidium* spp. The objective of this study was to determine the prevalence of *Giardia* and *Cryptosporidium* in dogs, cattle and goats in the Magude District of Maputo Province, Mozambique.

## Materials and methods

### Study area

**FIGURE 1:**
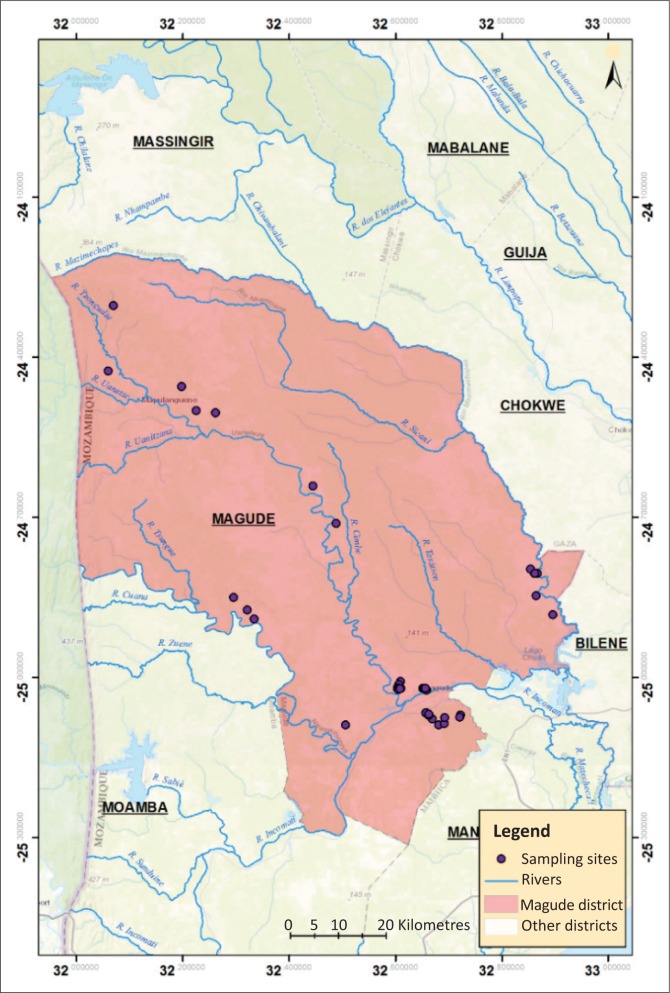
Map of Magude District and localities where dog and livestock samples were collected.

The study was conducted between February and September 2015 in the localities of the Magude District (Figure 1), Maputo Province, Mozambique, namely Magude Sede, Motaze, Mapulanguene, Panjane and Mahele. The climate in the study area is dry sub-tropical, with an annual temperature average of 22 °C – 24 °C and the annual rainfall average of 630 mm (MAE 2005). Livestock production and agriculture associated with animal traction are the main livelihoods of the community (INE 2009). In the district, cattle are reared extensively, goats are housed at night and released in the morning to the communal grazing areas, and dogs are bred freely with many of them trained to shepherd cattle in grazing areas.

### Sample collection and laboratory analysis

A total of 696 faecal samples were collected from the rectum of calves (*n* = 480), goat kids (*n* = 60) and dog pups (*n* = 156) all less than 7 months of age using a latex glove. Animals belonging to households pre-identified by the local veterinary technician were randomly selected at dip tanks during vaccination campaigns for calves, goats and dogs and a consent form was sought by each owner before sample collection. Sample consistence was classified as normal (soft to hard) or diarrheic (watery) and then transferred into tubes with caps which were labelled with individual details of each animal (animal species, age if possible and identification number) and transported in a cooler box to the Parasitology Laboratory, Faculty of Veterinary Medicine, Eduardo Mondlane University in Maputo, Mozambique, and kept at 4 °C until processed. A questionnaire was designed for dog owners and livestock farmers to collect information regarding animal husbandry, housing conditions, drinking water sources, feeding, treatment against parasitic infections and use of faeces in agricultural practices.

### Copromicroscopic analysis

Faecal samples were processed for the detection of *Giardia* and *Cryptosporidium* (oo)cysts using the Willis flotation technique as described by Ueno and Gonçalves (1998). Identification of (oo)cysts was performed using morphological characteristics as described by Taylor et al. (2007). To concentrate the (oo)cysts in faecal samples, the formol-ether technique was used as described by Cheesbrough (1987). The pellet obtained from the concentration was used to prepare thin smears that were stained by the modified Ziehl-Neelsen (mZN) method as described by Cheesbrough (1987) and observed under an optical microscope at 100× magnification for the presence or absence of *Cryptosporidium* oocysts. The remainder of the pellet was transferred to Eppendorf tubes and preserved at -20 °C for further processing for the detection of *Cryptosporidium* and *Giardia* by direct and indirect IF tests (DIF and IIF).

### Direct and indirect immunofluorescence

The DIF test was carried out using a kit (MERIFLUOR^®^ *Cryptosporidium* or *Giardia*; Meridian Diagnostic, United States [US]) according to the manufacturer’s specification. All dog and goat samples were analysed; however, because of the lack of resources, only 237 calf samples were randomly selected and analysed by this technique.

The IIF test was conducted in all faecal samples except those from goats because the secondary antibody was derived from goats. For this technique, 25 *µ*L of concentrated faeces by formol-ether method was transferred to an IF slide. This was left to dry for approximately 5 minutes and fixed with absolute methanol. Approximately 50 *µ*L of primary antibody (Anti-*Cryptosporidium parvum* mAb, Abnova and anti-*Giardia lamblia* pAb, Abnova, Europe) diluted in (3%) bovine serum albumin (BSA) in phosphate-buffered saline (PBS) (1:500) was added to the smear, incubated for 1 h in a wet chamber and then washed three times in PBS Tween-20 (0.05%). One drop of the secondary antibody coupled to fluorescein (Goat pAb to *Cryptosporidium parvum* oocyst and *Giardia* cysts or FITC, Abcam) diluted in 3% BSA in PBS (1:1000) was added to the smear, incubated in the dark for 30 min and washed three times to remove the excess of fluorescein. To obtain the optimal dilution of the secondary antibody, serial dilutions were made starting from 1:10. A mounting reagent was added to the slide, covered with a coverslip and observed under a fluorescence microscope (100×).

### Data analysis

A sample was considered positive if at least one cyst or oocyst of *Giardia* or *Cryptosporidium* was identified in the slide. The prevalence (%) was calculated as the number of positive samples divided by the total number of samples analysed multiplied by 100 (Thrusfield 1999). To analyse differences in the prevalence of *Giardia* and *Cryptosporidium* among the localities of the Magude District, a general linear multivariate model was applied in Statistical Package for the Social Sciences (SPSS) version 20.0 and *p* < 0.05 was considered to be statistically significant. MediCalc software was used to calculate the sensitivity and specificity of mZN and IIF with the DIF test used as a gold standard.

### Ethical considerations

This research was approved by the Scientific board of the Veterinary Faculty, Eduardo Mondlane University, Maputo, Mozambique.

## Results

*Giardia* cysts were detected in calves, young goats and pups and *Cryptosporidium* oocysts in calves and dogs, as shown in Table 1. Prevalence values of *Giardia* and *Cryptosporidium* were high according to the IIF test in pups (8.3%, CI: 8.0–8.5) (6.4%, CI: 6.1–6.6) and calves (8.1%, CI: 7.9–8.3) (4.7%, CI: 4.5–4.8), respectively. Following this technique, the prevalence of *Giardia* by the DIF test was 5.7% (CI: 5.4–5.9) in pups, 6.0% (CI: 5.8–6.2) in calves and *Cryptosporidium* by the mZN test in calves was 3.8% (CI: 3.6–3.9). In general, high prevalence values of *Giardia* spp. and *Cryptosporidium* spp. were recorded in the locality of Magude Sede for calves and dogs by IIF and the lower in the locality of Mahele for *Giardia*, and this association was significant (*p* < 0.05) as represented in Table 1. Neither of these parasites was detected in dogs from Motaze and Mahele. The prevalence rate of *Giardia* spp. in goat kids from Motaze and Magude Sede was the same (6.66%) and in other localities, no positive was detected in this animal species.

**TABLE 1:** Prevalence (%) of *Giardia* spp. and *Cryptosporidium* spp. in calves, goat kids and dog pups in different localities of the Magude District, Mozambique.

Locality	Calves					Goat kids	Pups					
	*Giardia*		*Cryptosporidium*			*Giardia*	*Giardia*			*Cryptosporidium*		
	DIF	IIF	mZN	DIF	IIF	DIF	Willis	DIF	IIF	mZN	DIF	IIF
Motaze	2 (0.8)	2 (0.4)	4 (0.8)	1 (0.4)	9 (1.8)	4 (6.66)	0	0	0	0	0	0
Mahele	0	1 (0.2)*	2 (0.4)	0	3 (0.6)	0	0	0	0	0	0	0
Mapulanguene	2 (0.8)	5 (1.0)	1 (0.2)	0	2 (0.4)	0	0	3 (1.9)	2 (1.3)	0	0	0
Panjane	0	6 (1.25)	3 (0.6)	0	5 (1.0)	0	0	0	1 (0.64)	0	0	1 (0.64)
Magude Sede	10 (4.2)	25 (5.2)	8 (1.6)	0	4 (0.8)	4 (6.66)	1 (0.64)	6 (3.8)	10 (6.4)	1 (0.64)	1 (0.64)	9 (5.8)

**Total**	**14 (6.0)**	**39 (8.1)**	**18 (3.8)**	**1 (0.4)**	**23 (4.7)**	**8 (13.3)**	**1 (0.64)**	**9 (5.7)**	**13 (8.3)**	**1 (0.64)**	**1 (0.64)**	**10 (6.4)**
**Total CI**	**5.8–6.2**	**7.9–8.3**	**3.6–3.9**	**0.35–0.44**	**4.5–4.8**	**12.5–14.0**	**0.52–0.67**	**5.4–5.9**	**8.0–8.5**	**0.52–0.67**	**0.52–0.67**	**6.1–6.6**
***N***	**237**	**480**	**480**	**237**	**480**	**60**	**156**			**156**		

DIF, direct immunofluorescence; IIF, indirect immunofluorescence; mZN, modified Ziehl-Neelsen; Willis, flotation technique; *N*, number; CI, confidence interval.

* , *p*-value based on general linear model multivariate (*p* = 0.001).

All samples collected in calves and goat kids had normal consistency, whilst three of the 156 samples from pups were diarrhoeic (1.9%) and from these, only one was positive for *Giardia* trophozoites (0.6%) by the Willis method.

The sensitivity and specificity of IIF, Willis and mZN are presented in Table 2. The mZN method showed high sensitivity (100%) and specificity (96.20% and 100%) to detect *Cryptosporidium* oocysts in calves and dogs, respectively. The IIF method showed high sensitivity and specificity to both parasites, the sensitivity ranging between 88.89% and 100%, and the specificity between 95.38% and 98.51% for *Giardia* spp.; with a sensitivity of 100% and specificity of 93.15% and 93.9% for *Cryptosporidium* spp.

**TABLE 2:** Sensitivity and specificity (95% confidence interval) of diagnostic tests used in the detection of *Cryptosporidium* oocysts and *Giardia* cysts in dogs and calves from the Magude District of Mozambique.

Test	*Cryptosporidium* spp.								*Giardia* spp.							
	Dogs				Calves				Dogs				Calves			
	Sensitivity		Specificity		Sensitivity		Specificity		Sensitivity		Specificity		Sensitivity		Specificity	
	Number	95% CI	Number	95% CI	Number	95% CI	Number	95% CI	Number	95% CI	Number	95% CI	Number	95% CI	Number	95% CI
Willis	0	0.0–97.5	100	95.1–100.00	0	0.0–84.2	100.00	93.4–100.0	0.00	0.0–33.63	98.46	91.7–99.96	0	0.0–11.94	100.00	98.17–100.00
mZN	100	2.5–100.0	100	95.1–100.00	100	15.8–100.0	96.20	93.2–98.5	–	-	–	-	–	-	–	1
IIF	100	25.5–100.0	93.15	84.7–97.74	100	15.8–100.0	93.90	89.9–96.6	88.89	51.7–99.70	95.38	87.1–99.00	100	88.1–100.00	98.51	95.70–99.69

IIF, indirect immunofluorescence; mZN, modified Ziehl-Neelsen; Willis, flotation technique; CI, confidence interval.

Other gastrointestinal helminths were observed in dog samples by the Willis method, namely *Ancylostoma* spp. with a prevalence of 60.3% (CI: 59.8–60.7) followed by *Toxocara* spp. (5.8% [CI: 5.6–5.9]), *Trichuris vulpis* (1.3% [CI: 1.2–1.4]), *Spirocerca lupi* 0.6% ([CI: 0.5–0.7]) and Taeniidae (1.9% [CI: 1.8–2.0]). In calves and goats, respectively, strongylid eggs were observed with prevalences of 50.8% (CI: 50.2–51.3) and 31.6% (CI: 30.5–32.6), *Eimeria* spp. with 17.5% (CI: 17.1–17.8) and 41.6% (CI: 40.4–42.7) and *Moniezia* spp. with 3.3% (CI: 3.1–3.4) and 11.6% (CI: 10.8–12.3).

## Discussion

The present study focussed on the diagnosis of *Giardia* spp. and *Cryptosporidium* spp. in livestock and dogs using copromicroscopical and immunological tests. This is the first study in Mozambique reporting parasites of the genus *Giardia* and *Cryptosporidium* in a mixed farming (cropping and livestock) rural community set-up.

The prevalence of 3.75% for *Cryptosporidium* spp. in calves by mZN in the present study is slightly higher than the prevalence reported in calves from 3 to 8 months (1.4%) and lower than the prevalence found in calves less than 3 months (16.6%) in a study made by Mtambo et al. (1997) in Tanzania using the same diagnostic technique. The categorisation of animals into two groups compared to this study may have caused this discrepancy. On the other hand, on most of the farms sampled in this study, the animals were kept housed, thus increasing the chances of transmission between animals (Taylor et al. 2007). The same factor may have contributed to the high prevalence of *Giardia* spp. (49%) and *Cryptosporidium* spp. (12%) found by Hamnes et al. (2006) in calves between 3 and 183 days from Norway using IF tests. In addition, animals from this Norwegian study were exposed to lower temperature conditions (between 3.6 °C and 14.0 °C) than from the animals of the Magude District (between 18 °C and 35 °C). High temperatures may reduce the viability of oocysts in the environment, whilst at temperatures near to 4 °C, the parasites can remain viable for more than 1 month (Adam 2014), hence increasing the risk of infection.

In general, the prevalence rates were higher by IIF compared to the DIF test for both parasites. Following the IIF method by mZN, the prevalence of *Cryptosporidium* in calves was also high and similar results were reported by Mtambo et al. (1992). The high sensitivity of IIF compared to mZN was also reported by Ortega-Mora et al. (1999) where same concentrated faecal samples of ewes were negative when analysed by mZN but positive by IIF.

Diarrhoea is a common clinical sign in animals infected by *Giardia* spp. and *Cryptosporidium* spp. (Dawson 2005; O’Donoghue 1995). The low incidence of animals with diarrhoea may suggest a low pathogenic significance of these parasitic infections in dog pups and calves in the Magude District. Besides the low pathogenic significance, other factors associated with the absence of clinical signs in positive animals are: (1) the phase of excretion of (oo)cysts because the peak coincides with the peak of animals with diarrhoea which is between the ages of 8–14 days for *Cryptosporidium* oocysts in cattle (Causapé et al. 2002; Olson et al. 2004) and between 2 and 4 weeks for *Giardia* (Geurden, Vercruysse & Claerebout 2010); (2) development of an immunological response with the advancing age of animals (Huber, Bomfim & Gomes 2005) and (3) the virulence of the genotype involved (Adam 2014). Although it was not possible to confirm the species and genotypes of *Cryptosporidium* and *Giardia* based on the techniques used, the zoonotic potential of these parasites should be taken into consideration, especially for *Cryptosporidium* spp. which is an opportunist in immune-compromised individuals such as those who are HIV-positive (Morgan et al. 2000). In Mozambique, Clavero et al. (1999) isolated *Cryptosporidium* spp. in HIV-infected humans.

The evaluation of sensitivity and specificity for Willis, mZN and IIF techniques compared to the DIF test showed a high sensitivity (100%) and specificity (96% – 100%) for the mZN test in the detection of *Cryptosporidium* infections. Results from our study indicate that the mZN technique is highly reliable in the diagnosis of *Cryptosporidium* spp. in faecal samples. Studies conducted by Zaglool et al. (2013) and Quílez et al. (1996) in the diagnosis of *Cryptosporidium* spp. by mZN test indicated low sensitivities (73.3% and 79.3%, respectively) and a specificity approximating to the present study (95% and 100%, respectively). The high sensitivity of the mZN test to detect *Cryptosporidium* spp. in this study can be attributed to the concentration of oocysts in faecal samples using the formalin-ether technique prior to analysis by subsequent tests. Salleh et al. (2014) demonstrated that the sensitivity of mZN can be improved by the application of concentration techniques.

In general, the sensitivity of IIF and mZN tests in the detection of *Cryptosporidium* spp. was similar (100%) in this study, these results were also similar to findings by Rimhanen-Finne et al. (2007). Despite the similarity of results, the choice of diagnostic technique often depends on the availability of resources, time and the objective to be reached (diagnosis of specific parasite or multiple parasites) (Chalmers 2014). In the IIF technique specific antibodies against antigens produced by the parasite are used making it easy to read owing to the incidence of the fluorescent light in oo(cysts), indicated especially in cases of a low intensity of infections (Robertson 2014). The disadvantage of mZN staining is that the oocysts may be easily confused with faecal debris that take up the stains (Casemore, Armstrong & Sands 1985). The efficiency of IIF compared to mZN was reported by Ortega-Mora et al. (1999) where the concentrated faecal samples of ewes were negative when analysed by mZN and positive by IIF.

*Ancylostoma* spp. (60.25%) and *Toxocara* spp. (5.76%) diagnosed in dogs of the Magude District are of zoonotic potential and the epidemiology of this parasite is mainly associated with the high biotic potential of females and with transmammary infection by which larvae are transmitted to the offspring from the bitch (Taylor et al. 2007; Urquhart et al. 1998). The lower prevalence of *Toxocara* spp. compared with *Ancylostoma* spp. can be justified by the possible presence of animals with larvae in somatic tissues in which, instead of the larvae developing, maturing and producing eggs, they remain dormant in different tissues, and thus, there is a reduction or absence of eggs in faeces (Taylor et al. 2007).

There is a need for additional studies aimed at applying molecular techniques to identify the genotypes and subtypes of *Giardia* and *Cryptosporidium* involved in order to determine their zoonotic potential and to adopt effective control and prevention measures.
